# Elucidating common pathogenic transcriptional networks in infective endocarditis and sepsis: integrated insights from biomarker discovery and single-cell RNA sequencing

**DOI:** 10.3389/fimmu.2023.1298041

**Published:** 2024-01-25

**Authors:** Chen Yi, Haoxiang Zhang, Jun Yang, Dongjuan Chen, Shaofeng Jiang

**Affiliations:** ^1^ Department of Biomedical Engineering, Nanchang Hang Kong University, Nanchang, Jiangxi, China; ^2^ Department of Laboratory Medicine, Maternal and Child Health Hospital of Hubei Province, Tongji Medical College, Huazhong University of Science and Technology, Wuhan, China

**Keywords:** infective endocarditis, sepsis, single-cell RNA sequencing, immune cells, IL-10

## Abstract

**Background:**

Infective Endocarditis (IE) and Sepsis are two closely related infectious diseases, yet their shared pathogenic mechanisms at the transcriptional level remain unclear. This research gap poses a barrier to the development of refined therapeutic strategies and drug innovation.

**Methods:**

This study employed a collaborative approach using both microarray data and single-cell RNA sequencing (scRNA-seq) data to identify biomarkers for IE and Sepsis. It also offered an in-depth analysis of the roles and regulatory patterns of immune cells in these diseases.

**Results:**

We successfully identified four key biomarkers correlated with IE and Sepsis, namely CD177, IRAK3, RNASE2, and S100A12. Further investigation revealed the central role of Th1 cells, B cells, T cells, and IL-10, among other immune cells and cytokines, in the pathogenesis of these conditions. Notably, the small molecule drug Matrine exhibited potential therapeutic effects by targeting IL-10. Additionally, we discovered two Sepsis subgroups with distinct inflammatory responses and therapeutic strategies, where CD177 demonstrated significant classification value. The reliability of CD177 as a biomarker was further validated through qRT-PCR experiments.

**Conclusion:**

This research not only paves the way for early diagnosis and treatment of IE and Sepsis but also underscores the importance of identifying shared pathogenic mechanisms and novel therapeutic targets at the transcriptional level. Despite limitations in data volume and experimental validation, these preliminary findings add new perspectives to our understanding of these complex diseases.

## Introduction

Infective Endocarditis (IE) and Sepsis are severe multi-systemic diseases, with the former being initiated by infection of the endocardial surface (1) and the latter being a life-threatening organ dysfunction caused by dysregulated host response to infection (2). Globally, it is estimated that 3-10 per 100,000 people are diagnosed with IE annually, with a rising incidence in some regions (3). Alarmingly, the global in-hospital mortality rate for IE is as high as 22%, reaching 45% in a 5-year span (3). IE frequently co-occurs with severe complications like sepsis. A study involving 894 IE patients indicated that 17.4% experienced septic shock during hospitalization (4). Additionally, an estimated 48.9 million people are diagnosed with sepsis worldwide annually, resulting in 11 million deaths, accounting for 19.7% of global mortality (5). These data underscore the prevalence and severity of IE and Sepsis and highlight the pressing need for in-depth studies to promote more precise and effective treatments.

In recent years, with the continuous advancement of medical technology, there has been a significant improvement in the understanding and treatment of IE and sepsis. Existing research has elucidated many key mechanisms and potential treatments for both diseases. For instance, in IE, S100A11 and AQP9 have been identified as potential biomarkers with diagnostic value (6), and significant associations have been noted with B-cell receptors, IL-17, and the NF-kappa B signaling pathway (7). Therapeutic drugs like ceftazidime-avibactam and aztreonam have been successful in treating IE patients (8). In sepsis, SCAMP5 shows promise for diagnosis biomarker (9), and the PI3K/Akt-HIF-1α pathway modulates immunological glycolysis, thereby controlling neutrophil function in sepsis patients (10). Timely antibiotic use also significantly improves the prognosis of septic shock patients (11). However, despite these breakthroughs, numerous challenges remain, particularly in elucidating disease mechanisms and developing novel therapeutic strategies.

Notably, although some IE patients present with severe sepsis complications (4), the shared transcriptional features and molecular signaling pathways between these diseases have not been thoroughly explored. Furthermore, the complexity lies in understanding how candidate genes shared between Infective Endocarditis (IE) and sepsis may influence the phenotypic outcomes in patients. Unraveling these phenotypic differences can provide crucial phenotypic information for personalized therapeutic strategies. Currently, antibiotics dominate the treatment landscape for both IE and sepsis but come with the risks of side effects and drug resistance. This has drawn attention to the development of small molecule drugs as effective supplements or potential alternatives, offering lower resistance and side effect profiles. Furthermore, the broad-spectrum antibiotic use in all suspected IE or sepsis patients not only increases the risk of unnecessary exposure in uninfected individuals but also consumes significant resources. This underscored the importance of early diagnosis and the development of small-molecule drugs.

**Figure 1:**
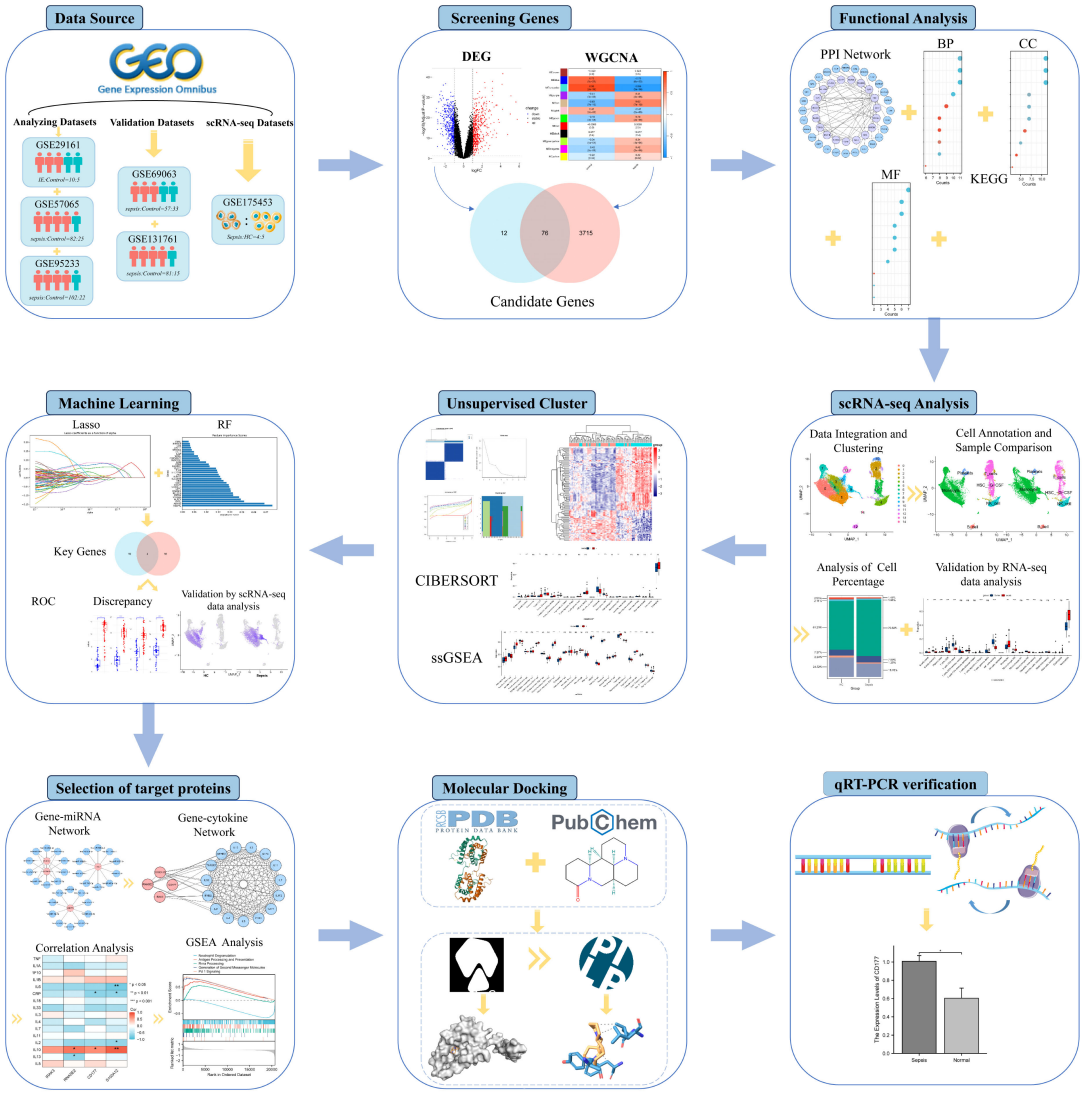
Research flowchart.

This study aims to uncover key biomarkers and signaling pathways in Infective Endocarditis (IE) and Sepsis, delving into their co-pathogenesis and phenotypic features, as outlined in **Figure 1**. Advanced bioinformatics techniques were employed for precise analysis of large-scale microarray data, successfully identifying key biomarkers and elucidating their functional roles in disease progression. Additionally, single-cell RNA sequencing (scRNA-seq) technology was employed to analyze differences in the expression patterns of immune cells between septic patients and healthy individuals. This enabled us to capture the expression patterns of immune cells at a single-cell resolution. This high-resolution approach further revealed specific functions and roles of various immune cell subtypes in sepsis development. We also focused on IL-10 as a potential therapeutic target, providing scientific rationale for the potential application of small molecule drugs in treating IE and Sepsis. Through validation studies on target binding efficacy and mechanisms of action, we explored small molecule drugs that could serve as antibiotic alternatives, hoping to mitigate the risks of side effects and antibiotic resistance. This research not only deepens our understanding of these complex multi-systemic diseases but also provides valuable insights and directions for future personalized and precise treatment plans.

## Methods

### Data source

The data used in this study were sourced from the Gene Expression Omnibus (GEO) database. We selected an microarray dataset related to Infective Endocarditis (IE): GSE29161, which includes 10 IE samples and 5 control samples and four microarray datasets related to Sepsis were chosen: GSE57065 with 82 sepsis samples and 25 control samples; GSE69063 with 57 sepsis samples and 33 control samples; GSE95233 with 102 sepsis samples and 22 control samples; and GSE131761 with 81 sepsis samples and 15 control samples. A single-cell RNA sequencing (scRNA-seq) dataset related to Sepsis, GSE175453, was also included, featuring 4 sepsis samples and 5 healthy control (HC) samples; and a sepsis RNA-seq data GSE185263, which comprises 348 septic samples and 44 healthy samples. The analysis datasets were designated as the IE dataset GSE29161, two sepsis microarray datasets with larger sample sizes (GSE57065 and GSE95233), and the scRNA-seq data GSE175453. The validation datasets were selected as two sepsis microarray datasets with relatively smaller sample sizes (GSE69063 and GSE131761) and the RNA-seq data GSE185263. Platform files corresponding to each microarray dataset were downloaded from the GEO database. Annotation and normalization of each dataset were performed using R software for subsequent analysis or validation.

### Identification of differentially expressed genes

The “limma” package was employed to identify DEGs between control samples and IE or Sepsis samples, based on the following criteria: |log_2_(Fold-Change)|>1,adjPvalue<0.05. Heatmaps and volcano plots of the differential analysis results were generated using the “pheatmap” and “ggplot2” packages, respectively.

### Weighted gene co-expression network analysis

The “WGCNA” package was utilized to construct a co-expression network. Initially, clustering was performed on the samples to assess the presence of any noticeable outliers. Subsequently, a “soft” thresholding power (β) was selected based on the scale-free topology criterion to achieve a scale-free network topology. Furthermore, an adjacency matrix was derived from the topological overlap map (TOM), and stable gene modules were identified using the dynamic tree-cutting algorithm. Modules with a feature factor greater than 0.1 were merged to enhance module stability. Based on the module eigengene matrix and sample traits, correlations between modules and traits were calculated and visualized.

### Construction of protein-protein interaction network and enrichment analysis

The intersection of all genes identified through differential analysis and WGCNA was taken to obtain candidate genes for further analysis. Protein interactions were predicted using the STRING database, retaining interactions between two proteins with a medium confidence score (confidence score≥0.4). The PPI network was constructed using Cytoscape. Functional annotation of the candidate genes was carried out using the “clusterProfiler” package in R, which included GO functional annotation and KEGG pathway enrichment analysis. The criterion for determining significant enrichment was P-value (adjP-value) <0.05.

### scRNA-seq data analysis

Use the Seurat package to process all samples from GSE175453. Firstly, perform quality filtering on the samples using different standards (**Supplementary Table 1**), and then merge them into a Seurat object. Log-normalize the merged data (scale factor = 10000), followed by feature selection and standardization. After performing Principal Component Analysis (PCA), correct batch effects between different samples using the Harmony package. Subsequently, identify significant principal components through JackStraw analysis, cluster cells using FindNeighbors and FindClusters, and select an appropriate resolution parameter. Finally, perform dimensionality reduction through Uniform Manifold Approximation and Projection (UMAP) for visualization purposes. Subsequently, the “SingleR” package was used to compare the transcriptomic expression of each cell with the “HumanPrimaryCellAtlasData” reference dataset from the Celldex index (12), annotating each cell using cellular terms derived from this reference dataset.

### Immune cell infiltration-related analysis

The abundance of immune cells in different samples was assessed using the CIBERSORT algorithm. Additionally, single-sample gene set enrichment analysis (ssGSEA) was performed using the “GSEAbase” package based on the expression levels of 29 immune-related markers. Concurrently, the Wilcoxon rank-sum test was employed to calculate differentially enriched immune cells between samples.

### Unsupervised clustering

For the case of Sepsis, represent ed by the GSE57065 dataset, unsupervised clustering analysis was performed on the candidate genes. The number and robustness of clusters were evaluated using the “ConsensusClusterPlus” package. The k-means clustering method was applied with 50 iterations, and each validation was carried out using 80% of the samples to ensure the stability of the clustering. The “limma” package was used to assess the gene expression differences between different clusters.

### Key gene identification via multiple machine learning

Two machine learning algorithms, the Least Absolute Shrinkage and Selection Operator (LASSO) and Random Forest (RF), were employed to select key genes. In the LASSO analysis, 5-fold cross-validation was used to optimize the selection of the regularization parameter (α). The importance of genes was determined based on their coefficients and visualized using bar graphs. The RF algorithm utilized a Bayesian optimization strategy for selecting the best hyperparameters for model training. Gene influence was ascertained through feature importance scores, which were graphically represented using bar charts to intuitively display the results. Genes commonly selected by both algorithms were considered as diagnostic genes or key cluster genes.

### mRNA-miRNA interaction and correlation analysis

Predicting potential regulatory miRNAs for diagnostic genes from the TargetScan database (www.targetscan.org), the top 10 miRNAs were selected based on the context++ score percentile. Visualization was performed using Cytoscape software. Genes related to inflammatory cytokines were retrieved from the Uniprot database. Pearson’s method was used to calculate the correlation between diagnostic genes and inflammatory cytokines. A heatmap was generated to visualize the relationships between diagnostic genes and inflammatory cytokines.

### Molecular docking

The receptor protein structures were downloaded from the Protein Data Bank (PDB), and the 3D structures of ligand small molecules were downloaded from the PubChem database. Prior to molecular docking, water molecules in the protein structures were removed using Pymol software.Add Hydrogens, charge calculation, and atom type assignments were performed using AutoDockTools software (v1.5.7). Molecular docking was carried out using AutoDock Vina (13), where the processed structures were subjected to simulations to analyze the binding characteristics of the small molecule ligands with the target proteins. The molecular conformation with the highest binding affinity from the docking results was retained. Subsequent analysis of the ligand-protein interactions was conducted using the Protein-Ligand Interaction Profiler (PLIP) web tool (14). Finally, the interactions were visualized using Pymol.

### Real-time quantitative polymerase chain reaction

To validate the reliability of the biomarkers identified in this study, real-time Quantitative Polymerase Chain Reaction (qRT-PCR) was performed on samples obtained from 5 sepsis patients and 5 healthy controls. The acquisition of these samples was approved by the Hubei Provincial Maternal and Child Health Hospital, and informed consent was obtained from each participant involved in the study. Total RNA from cells or tissues was extracted using Trizol reagent (Ambion, China), and qRT-PCR was conducted using the SYBR FAST qPCR Master Mix (KAPA Biosystems, China). GAPDH was used as an internal control, and the relative expression levels were calculated using the formula 2^(-ΔΔCt). The primer sequences are listed in **Supplementary Table 1**.

### Statistical analysis

R (v 4.3.0) was used for all statistical analysis in this study. And Python (v 3.11) was used for machine learning. P < 0.05 was considered statistically significant, and the Pearson coefficient were used to assess the correlation. In addition, binding energy was used to evaluate the effectiveness of the molecular docking. The biological images used in this study were sourced from Smart-Servier Medical Art (https://smart.servier.com/).

## Results

### Data preprocessing

Due to the subsequent analysis not merging different microarray datasets, batch effects between different datasets are not taken into consideration. Perform quantile normalization on the microarray datasets using the limma package. As depicted in the box plots (**Supplementary Figure 1**) normalization effectively mitigated technical variations. In cases where different probes matched the same gene symbol, the first occurrence of the probe was retained as the gene’s expression value. Notably, datasets GSE57065, GSE69063 and GSE95233 encompassed samples from the same sepsis patients collected at various time points. Principal Component Analysis (PCA) applied to these three datasets (**Supplementary Figure 2**) revealed substantial similarity in the two-dimensional projection of samples collected at different time points. This observation underscores the high consistency of these datasets in terms of their features. Therefore, for subsequent analyses, data collected at different time points after diagnosis from the same dataset were merged to serve as representative data for sepsis.

For the RNA-seq dataset GSE185263, raw count data was retained for genes with counts >1. Normalization was performed using the DESeq2 package, and conversion of ENSEMBL IDs to Gene Symbols was accomplished using the org.Hs.eg.db package.

### Identification of DEGs between patient and normal

**Figure 2:**
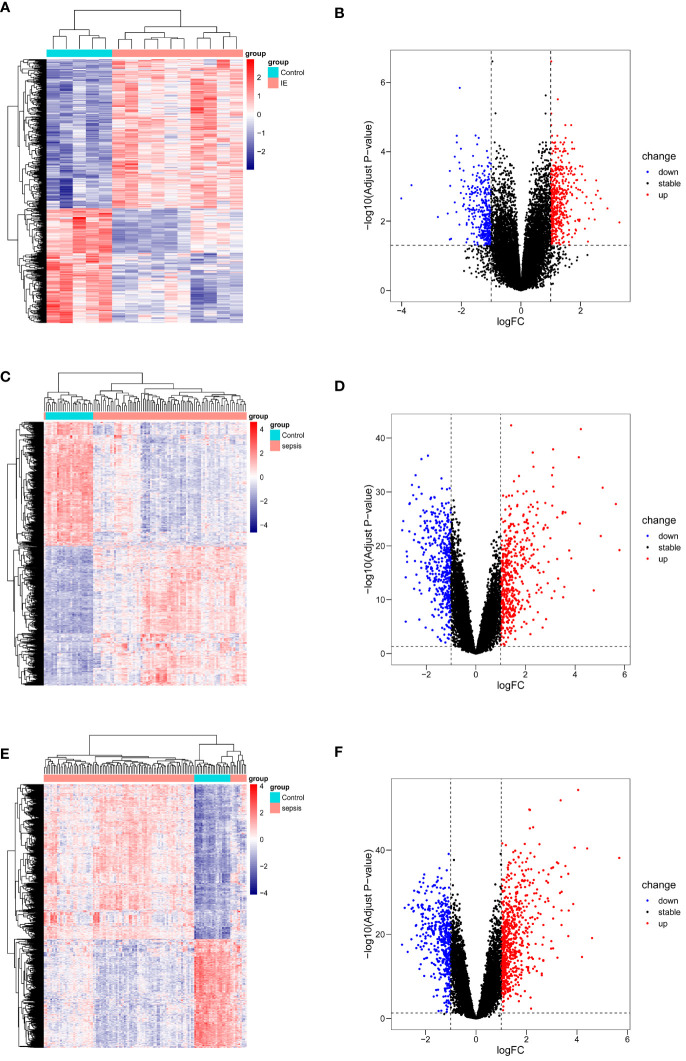
DEGs visualization of datasets. **(A, B)** DEGs from GSE29161. **(C, D)** DEGs from GSE57065. **(E, F)** DEGs from GSE95233.

The differential analysis results for identifying DEGs with the same threshold across the datasets (GSE29161, GSE57065, GSE95233) are shown in **Figure 2**, where the heatmap displays all identified DEGs. Specifically, for the comparison between IE samples and normal samples, 816 DEGs were identified in GSE29161 (355 downregulated and 461 upregulated). For the comparison between sepsis samples and normal samples, 914 DEGs were identified in GSE57065 (429 downregulated and 485 upregulated), and 1113 DEGs were identified in GSE95233 (460 downregulated and 653 upregulated).

### Identification of co-expressed gene modules associated with sepsis

**Figure 3:**
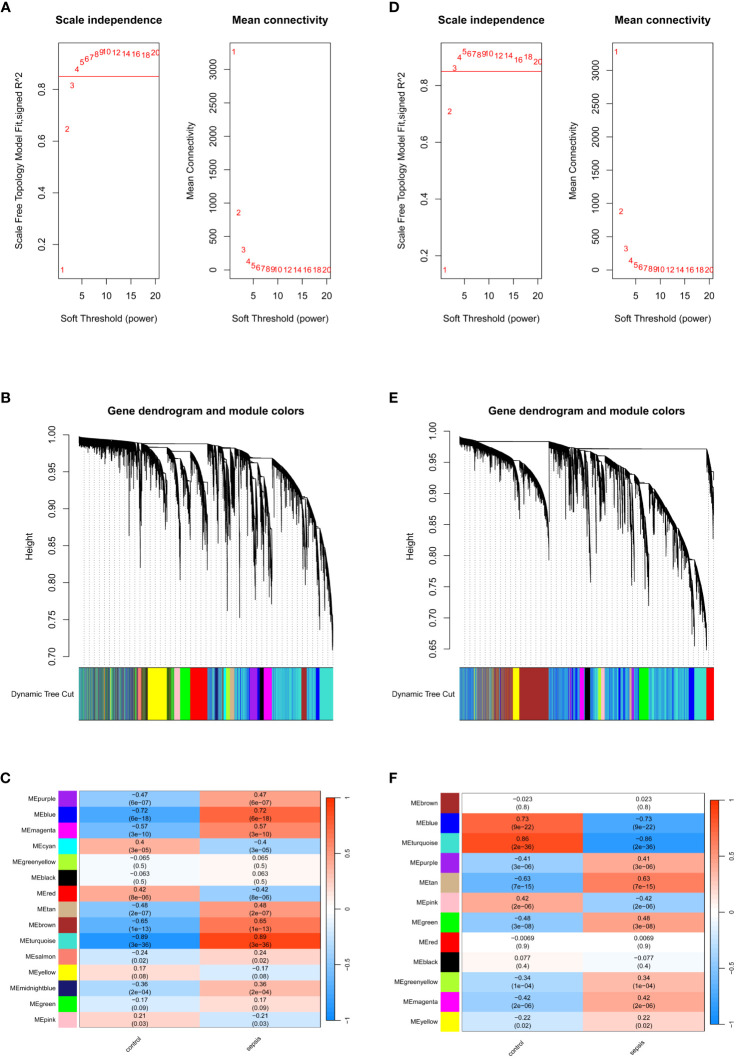
Identification of module genes via WGCNA. **(A)** Soft threshold analysis in GSE57065. **(B)** Clustering dendrogram and merging of the gene co-expression modules represented by different colors in GSE57065. **(C)** Heatmap of the module–trait relationship in GSE57065. **(D)** Soft threshold analysis in GSE95233. **(E)** Clustering dendrogram and merging of the gene co-expression modules represented by different colors in GSE95233. **(F)** Heatmap of the module-trait relationship in GSE95233.

WGCNA analysis was performed on the GSE57065 and GSE95233 datasets to identify gene modules associated with the sepsis phenotype. In GSE57065, no data were excluded due to missing values, and cluster analysis excluded three significant outliers. Soft-thresholding analysis indicated that when β=4 (scale-free R^2 =^ 0.85), the correlation between genes was most consistent with a scale-free distribution (**Figure 3A**). With the minimum module size set at 200, 15 modules were successfully identified by merging similar modules (**Figures 3B, C**). Among them, the MEturquoise module exhibited the highest correlation with sepsis(r=0.89,p=3e-36), containing 6972 genes. Similarly, in GSE95233, no data were excluded due to missing values, and two significant outliers were excluded through clustering analysis. β=3 was chosen as the optimal soft-thresholding value (**Figure 3D**). With the minimum module size set at 150, 12 modules were identified by merging similar modules (**Figures 3E, F**). Among these, the MEturquoise module showed the strongest association with the sepsis phenotype(r=-0.86,p=2e-36), containing 8375 genes.

### Functional analysis of DEGs and key module genes

**Figure 4:**
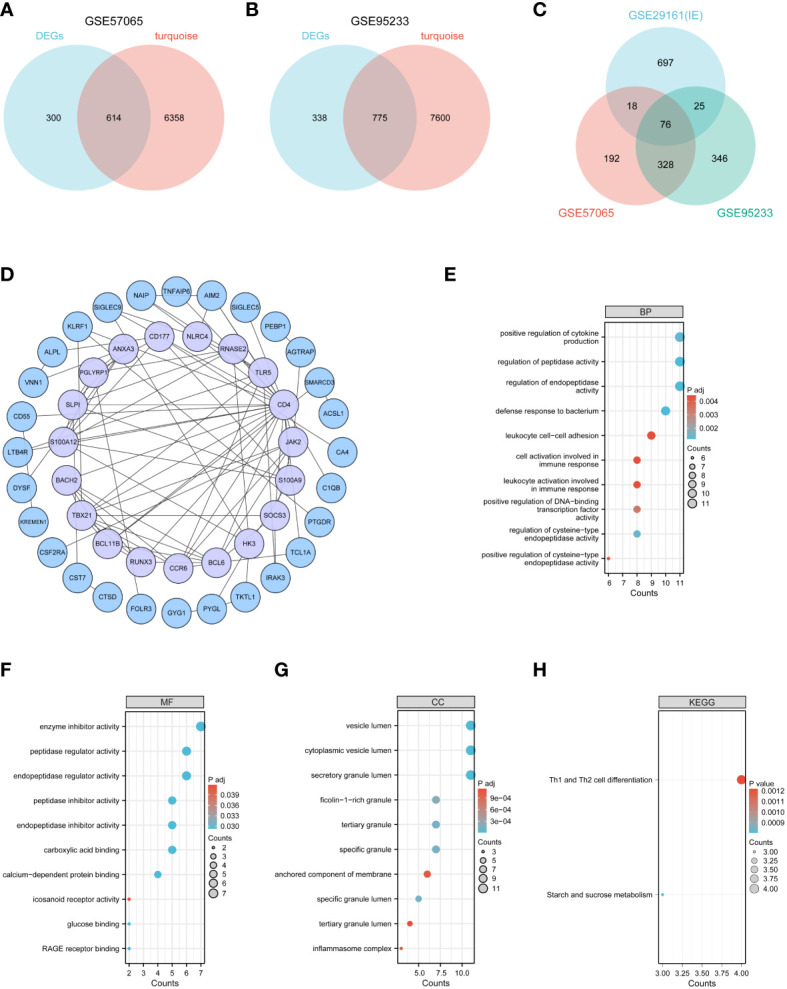
Functional analysis of common genes between DEGs and key modular genes. **(A–C)** Venn diagram of common genes between DEGs and key modular genes. **(D)** The PPI network among candidate genes. **(E–G)** GO analysis. **(H)** KEGG analysis.

Intersecting the differentially expressed genes (DEGs) and key module genes from GSE57065 and GSE95233 separately revealed 614 and 775 commonly expressed DEGs (**Figures 4A, B**). Taking these two gene sets as the distinctive genes for sepsis, their intersection with DEGs from infective endocarditis (IE) identified 76 candidate genes (**Figure 4C**). Functional analysis was performed on these candidate genes to explore their biological roles in IE and sepsis, aiming to identify potential shared pathogenic mechanisms between the two conditions.

To further investigate the functions of these candidate genes, a Protein-Protein Interaction (PPI) network was constructed (**Figure 4D**), comprising 47 nodes and 93 edges representing interactions between genes. GO and KEGG enrichment analyses were performed on the candidate genes. The results showed that these genes were primarily involved in immune and inflammatory functions: in terms of Biological Processes (BP), they were primarily focused on regulating protease activity, defense responses to bacteria, and cellular activation in immune responses (**Figure 4E**); in terms of Cellular Components (CC), they were mainly located in vesicle lumen and granules or complexes in immune cells (**Figure 4F**); in terms of Molecular Functions (MF), they primarily involved molecular binding, regulation of protease activity, and receptor activity (**Figure 4G**); and in KEGG pathway analysis, they were mainly involved in Th1 and Th2 cell differentiation (**Figure 4H**).

### scRNA-seq data analysis

**Figure 5:**
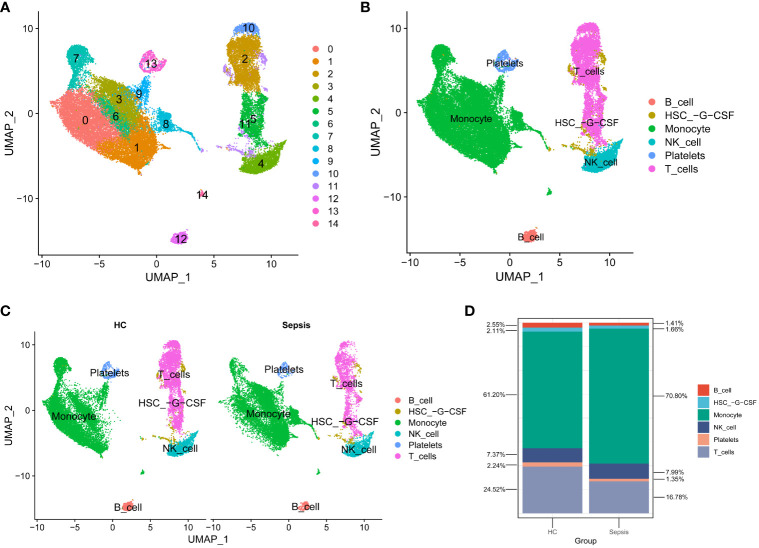
scRNA-seq data analysis. **(A)** Cells were categorized into 15 clusters. **(B)** The 15 clusters were further assigned to 6 known cell lineages. **(C)** Comparison of Cell Distribution between Healthy Control and Sepsis Samples. **(D)** Percentages of Cells in Healthy Control and Sepsis Samples.

The scRNA-seq data analysis incorporated 4 sepsis samples and 5 healthy controls (HC). After quality filtering (**Supplementary Table 2**), a total of 32,469 cells were selected. After JackStraw analysis, the top 17 principal components were selected. Cell-to-cell neighbor relationships were then calculated with a resolution of 0.5, followed by dimensionality reduction and projection using UMAP. The UMAP dimensionality reduction revealed that these cells were classified into 15 clusters (**Figure 5A**). These 15 clusters were further assigned to 6 known cell lineages (**Figure 5B**). The proportion of cells in each lineage was calculated separately for sepsis and HC samples, showing a relative enrichment of monocytes in sepsis samples, while B cells and T cells were relatively sparse (**Figures 5C, D**).

### Identification of sepsis clusters

**Figure 6:**
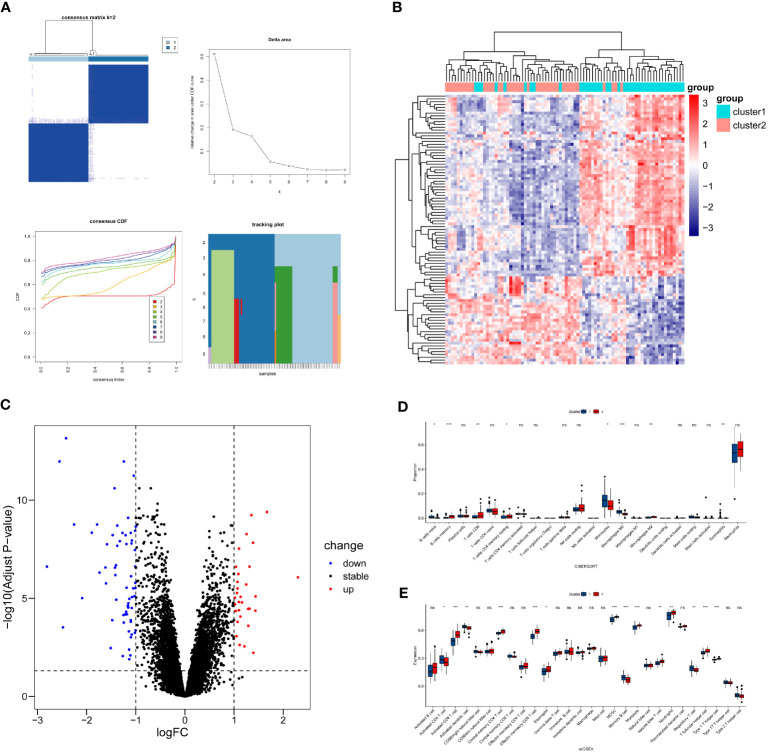
Differential expression and immune infiltration status between sepsis clusters. **(A)** Unsupervised clustering results, left top - Consensus Matrix(visualizes the frequency with which data points are assigned to the same cluster across multiple clustering runs), left bottom - Consensus Cumulative Distribution Function(CCDF, measures the degree of consistency in the assignment of each data point to different clusters), right top - Delta Area(indicates the change in slope of the CCDF curve), right bottom - Tracking Plot(depicts the dynamic changes of data points across different clusters), based on these visualizations, the optimal number of clusters is 2. **(B, C)** Visualization of DEGs between sepsis clusters. **(D)** Differences in abundance of immune cells from blood samples. **(E)** Differences in expression levels of immune cells from blood samples. * P<0.05, ** P<0.01, *** P<0.001, ns: not significance.

To investigate whether candidate genes influence the phenotypic outcomes in septic patients, consensus clustering was performed based on the expression patterns of 76 candidate genes in the sepsis samples from GSE57065. According to the results of Consensus Clustering, the optimal number of clusters for sepsis samples in GSE57065 was determined to be 2 (k value=2) (**Figure 6A**). Based on the criteria of |log2(Fold-Change)|>1 and adjPvalue<0.05, 95 differentially expressed genes (DEGs) were identified between the two sepsis clusters, including 64 downregulated genes and 31 upregulated genes (**Figures 6B, C**).

Due to the fact that GSE57065 consists of samples derived from blood, the immune cell analysis involves immune cells obtained from blood samples. Utilize the Cibersort method in conjunction with the LM22 feature matrix to quantify the abundance of 22 immune cell types in two sepsis clusters (**Figure 6D**). The results revealed that B cell naïve, Monocytes, Macrophages M0, and Eosinophils were more abundant in sepsis cluster 1 (P<0.05), while B cell memory, T cells CD8, T cells CD4 memory resting, and Macrophages M2 were more abundant in sepsis cluster 2 (P<0.05).

The ssGSEA analysis was employed to assess the expression levels of 28 types of immune cells in the two sepsis clusters (**Figure 6E**). The analysis indicated that Activated CD4 T cell, Activated dendritic cell, Memory B cell, and Regulatory T cell were more significantly expressed in sepsis cluster 1 (P<0.05). Conversely, Activated CD8 T cell, Central memory CD4 T cell, Effector memory CD8 T cell, Eosinophil, MDSC, Monocyte, Natural killer T cell, Neutrophil, T follicular helper cell, and Type 1 T helper cell showed more significant expression in sepsis cluster 2 (P<0.05).

### Key biomarker identification

**Figure 7:**
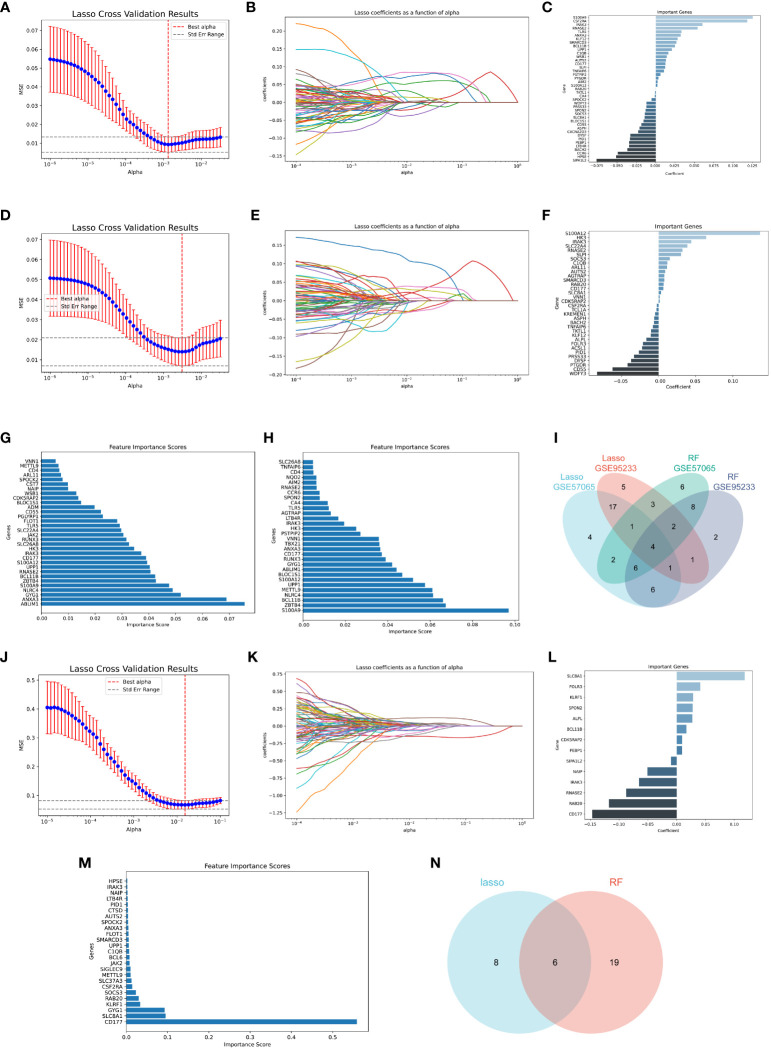
Machine learning screening for key biomarkers. **(A–C)** Screening of key biomarkers from GSE57065 using Lasso modeling. **(D–F)** Screening of key biomarkers from GSE95233 using Lasso modeling. **(G)** RF algorithm shows importance of genes from GSE57065. **(H)** RF algorithm shows importance of genes from GSE95233. **(I)** Co-diagnostic genes. **(J–L)** Screening of key biomarkers from two sepsis clusters samples using Lasso modeling. **(M)** RF algorithm shows importance of genes from two sepsis clusters samples. **(N)** Key cluster genes.

To further identify the key biomarkers associated with Infective Endocarditis (IE) and Sepsis, machine learning algorithms were applied to screen critical diagnostic genes from 76 genes in the sepsis datasets (GSE57065, GSE95233). Lasso regression algorithm identified 41 genes in GSE57065 (**Figures 7A–C**) and 34 genes in GSE95233 (**Figures 7D–F**). The Random Forest (RF) algorithm, based on gene importance, determined 32 genes in GSE57065 (**Figure 7G**) and 30 genes in GSE95233 (**Figure 7H**). Combining the results from both machine learning algorithms across the two datasets, four common diagnostic genes (IRAK3, CD177, RNASE2, S100A12) were finally selected (**Figure 7I**).

Additionally, in the quest for biomarkers that could effectively delineate different sepsis clusters, the same two machine learning algorithms were employed to screen genes from the sepsis cluster samples. Lasso regression identified 14 genes (**Figures 7J–L**), while the RF algorithm determined 25 genes (**Figure 7M**). Among the results from both algorithms, six key cluster-specific genes (SLC8A1, KLRF1, NAIP, IRAK3, RAB20, CD177) were commonly identified (**Figure 7N**).

### Evaluation of diagnostic or clustering potency of key biomarkers

**Figure 8:**
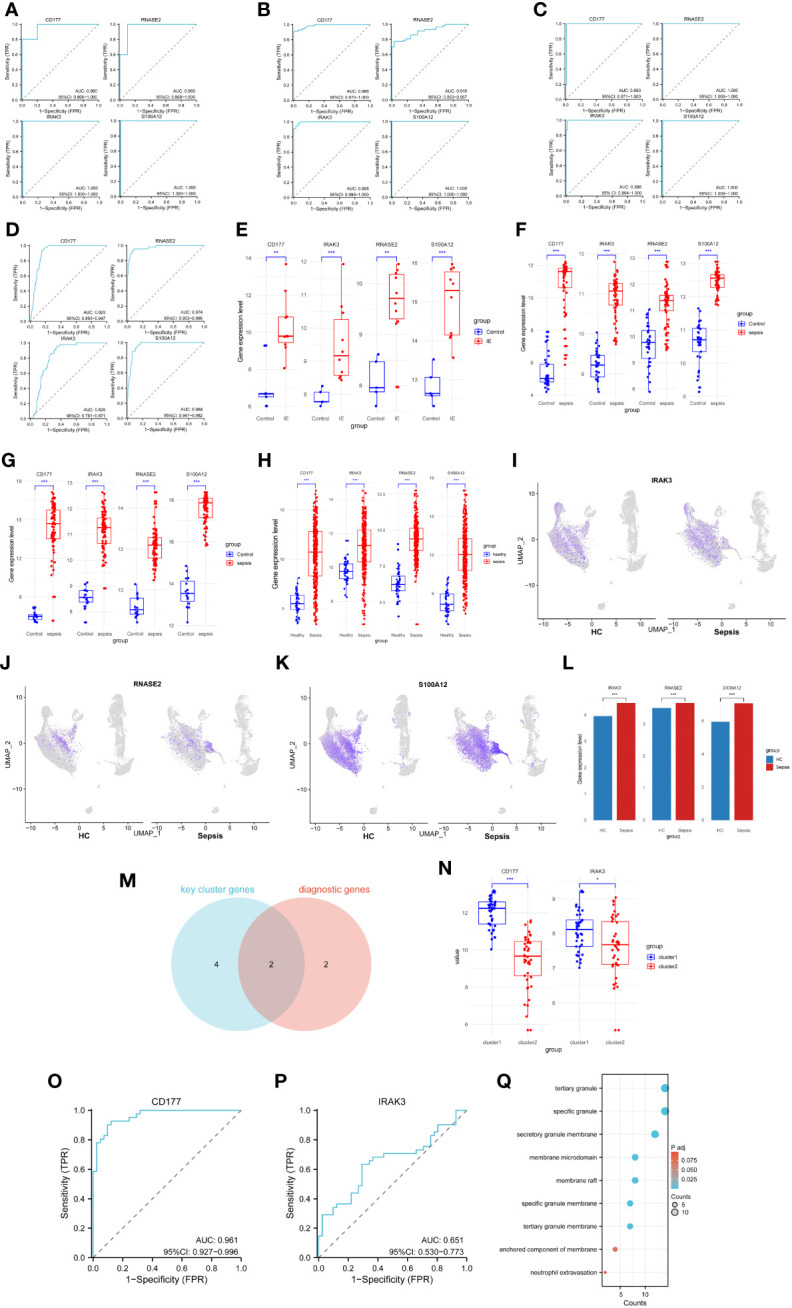
Capacity assessment of key biomarkers. **(A–D)** ROC curve for the Infective Endocarditis dataset **(A)**, two sepsis validation datasets (GSE69063 - B, GSE131761 - C) and sepsis RNA-seq data(GSE185263 - D), left top - CD177, left bottom - IRAK3, right top - RNASE2, right bottom - S100A12. **(E)** Expression levels of diagnostic genes for GSE29161. **(F)** Expression levels of diagnostic genes for GSE69063. **(G)** Expression levels of diagnostic genes for GSE131761. **(H)** Expression levels of diagnostic genes for GSE185263. **(I)** Cellular distribution of IRAK3. **(J)** Cellular distribution of RNASE2. **(K)** Cellular distribution of S100A12. **(L)** Differences in expression levels of key biomarkers for scRNA-seq. **(M)** Common genes for diagnostic and cluster genes. **(N)** Expression levels of cluster genes for two sepsis clusters samples. **(O)** ROC curve for sepsis clusters samples, based on CD177. **(P)** ROC curve for sepsis clusters samples, based on IRAK3. **(G)** Pathways associated with the action of CD177 in the enrichment results of two sepsis cluster DEGs. * P<0.05, ** P<0.01, *** P<0.001, ns: not significance.

To validate the diagnostic potential of the selected genes for Infective Endocarditis (IE) and Sepsis, Receiver Operating Characteristic (ROC) analysis was employed to build predictive models. By calculating the True Positive Rate (TPR), False Positive Rate (FPR), and the Area Under the Curve (AUC), ROC curves were plotted for visualization. The results indicated that the four diagnostic genes exhibited excellent diagnostic performance in the IE dataset GSE29161 (**Figure 8A**), two sepsis validation datasets, GSE69063 (**Figure 8B**), GSE131761 (**Figure 8C**), sepsis RNA-seq GSE185263 (**Figure 8D**) and two sepsis analysis datasets (GSE57065, GSE95233) (**Supplementary Figure 3A–H**), within the 95% confidence interval, the lowest AUC also reached 0.781. Additionally, by comparing the expression profiles between disease samples and control samples, it was found that the expression levels of CD177, RNASE2, IRAK3, and S100A12 were significantly elevated in both IE and sepsis samples (**Figures 8E-H**; **Supplementary Figure 3I–J**).

To further validate the diagnostic potency of these biomarkers, scRNA-seq data analysis was performed. Expression of three biomarkers (IRAK3, RNASE2, S100A12) was visualized in both HC and sepsis samples (**Figures 8I–K**). The expression matrices of scRNA-seq were extracted, and the Wilcoxon rank-sum test was used to analyze the expression differences between HC and sepsis samples. The results demonstrated a significant upregulation of these biomarkers in the sepsis samples (**Figure 8L**).

Moreover, to ascertain whether these diagnostic genes also possessed good clustering capabilities across different sepsis groups, an intersection was made between the machine learning-selected diagnostic genes and key cluster genes, resulting in two intersecting genes (IRAK3, CD177) (**Figure 8M**). ROC analysis was performed on these two intersecting genes, and expression level comparisons were conducted among different clusters to identify the discriminatory capability of the intersecting genes in distinguishing sepsis clusters. The results revealed that CD177 showed a significantly elevated expression level in Cluster 1 (**Figure 8N**) and demonstrated excellent clustering ability (**Figure 8O**). In contrast, although IRAK3 showed significant expression differences between the two clusters (**Figure 8N**), its clustering performance was less effective (**Figure 8P**). In addition, CD177 was found to be the only gene present among the 95 DEGs in the two sepsis clusters. Despite conducting enrichment analysis on these 95 DEGs, no significant pathway enrichment was observed in the KEGG analysis. Results from the GO analysis, focusing on pathways related to the action of CD177, were extracted and presented in a bubble chart (**Figure 8Q**). Among them, the pathway ‘Neutrophil Extravasation’ is related to physiological processes, while the remaining pathways are associated with cellular components. These pathways primarily function in the inflammation and immune response processes (Neutrophil Extravasation, the process of neutrophils moving from the blood vessels to infected or damaged tissues), and the structural components related to immune cells, inflammatory response, and cell signaling. (specific granule, tertiary granule, secretory granule membrane, tertiary granule membrane, specific granule membrane, membrane raft, membrane microdomain, anchored component of membrane).

### Target proteins for treatment

**Figure 9:**
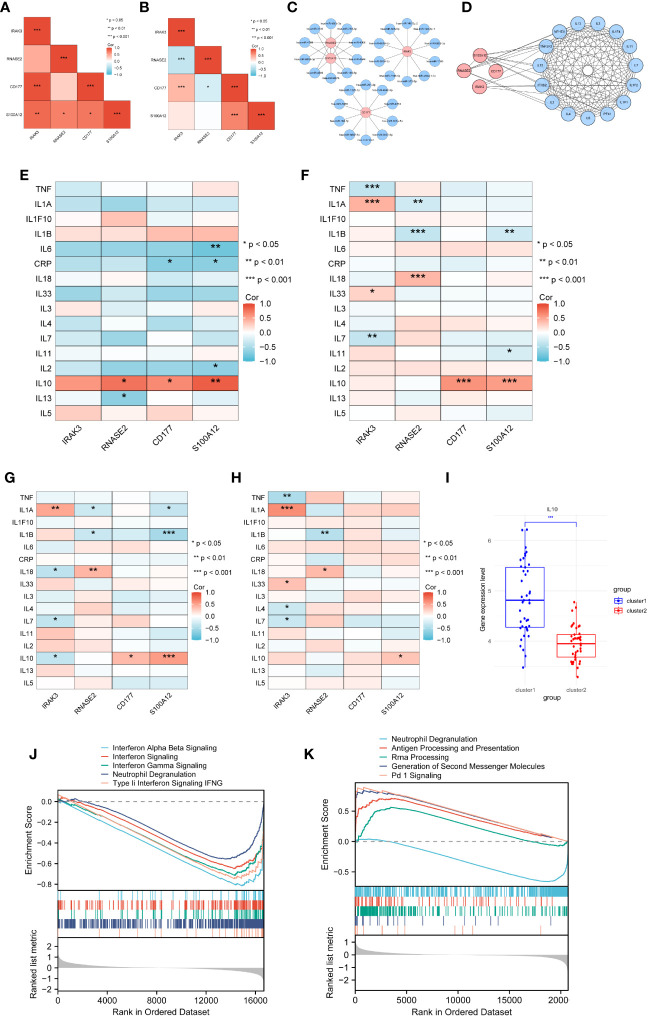
Selection of target proteins. **(A)** Correlation among diagnostic genes based on GSE29161. **(B)** Correlation among diagnostic genes based on GSE57065. **(C)** mRNA-miRNA network. **(D)** PPI network for diagnostic genes and inflammatory cytokines. **(E)** Correlation between diagnostic genes and inflammatory cytokines in GSE29161. **(F)** Correlation between diagnostic genes and inflammatory cytokines in sepsis samples from GSE5706. **(G–H)** Correlation between diagnostic genes and inflammatory cytokines in sepsis clusters sample(G-sepsis cluster1,H- sepsis cluster2). **(I)** Expression levels of IL10 for sepsis clusters samples. **(J)** GSEA analysis of GSE29161. **(K)** GSEA analysis of GSE57065. * P<0.05, ** P<0.01, *** P<0.001, ns: not significance.

The correlation between four genes was calculated using the Pearson method based on GSE29161 (**Figure 9A**), GSE57065 (**Figure 9B**), GSE69063 (**Supplementary Figure 4A**), GSE95233 (**Supplementary Figure 4B**), GSE131761 (**Supplementary Figure 4C**), and GSE185263 (**Supplementary Figure 4D**), revealing tight associations among them. Utilizing the TargetScan database, an mRNA-miRNA interaction network was constructed for diagnostic genes (**Figure 9C**). The network comprises 34 nodes and 40 edges, with the relationships between genes and miRNAs detailed in **Table 1**. These miRNAs play a crucial role in immune response, inflammatory response, cell cycle regulation, and the development of tumors.

**Table 1:** mRNA-miRNA.

Gene symbol	pp
CD177	hsa-miR-6807-5p, hsa-miR-194-3p, hsa-miR-8088, hsa-miR-1225-3p, hsa-miR-204-3p, hsa-miR-4646-5p, hsa-miR-4314, hsa-miR-323a-5p, hsa-miR-3620-5p, hsa-miR-1587
IRAK3	hsa-miR-450a-1-3p, hsa-miR-370-5p, hsa-miR-7106-5p, hsa-miR-3934-3p, hsa-miR-6814-5p, hsa-miR-505-5p, hsa-miR-140-3p.2, hsa-miR-124-3p.1, hsa-miR-6822-3p, hsa-miR-1193
RNASE2	hsa-miR-4689, hsa-miR-6858-5p, hsa-miR-4765, hsa-miR-3139, hsa-miR-450b-3p, hsa-miR-769-3p, hsa-miR-4306, hsa-miR-4644, hsa-miR-185-5p, hsa-miR-8076
S100A12	hsa-miR-4689, hsa-miR-6858-5p, hsa-miR-4765, hsa-miR-3139, hsa-miR-450b-3p, hsa-miR-769-3p, hsa-miR-4306, hsa-miR-4644, hsa-miR-185-5p, hsa-miR-8076

Twenty-six genes related to inflammatory cytokines were retrieved from the Uniprot database. A PPI (Protein-Protein Interaction) network was constructed between diagnostic genes and these inflammatory cytokines, consisting of 19 nodes and 126 edges representing their interactions (**Figure 9D**). Through analysis of IE and sepsis samples, the correlation between diagnostic genes and inflammatory cytokines was calculated. Sixteen of these genes being expressed within microarray data, while 14 inflammatory cttokines were expressed within RNA-seq data. The results indicate that, based on the IE dataset GSE29161 (**Figure 9E**) as well as the sepsis datasets GSE57065 (**Figure 9F**), GSE69063 (**Supplementary Figure 4E**), GSE95233 (**Supplementary Figure 4F**), GSE131761 (**Supplementary Figure 4G**) and GSE185263 (**Supplementary Figure 4H**), IL10 shows a significant correlation with diagnostic genes.

Further analysis of the two sepsis clusters revealed inconsistent correlations of IL10 within the clusters (**Figures 9G, H**). Comparing the expression profiles of IL10 between the two sepsis clusters revealed that IL10 is significantly upregulated in sepsis cluster 1 (**Figure 9I**).

Based on the median expression levels of IL10, the IE (GSE29161) and sepsis datasets (GSE57065) were divided into high IL10 expression and low IL10 expression groups. Gene set enrichment analysis (GSEA) was subsequently performed to identify biological activities and pathways differentially expressed at varying levels of IL10. The results indicated that in the IE dataset, labels related to interferon signaling and cellular granule release were decreased (**Figure 9J**). In the sepsis dataset, levels of labels related to cellular granule release were decreased, while antigen processing and presentation pathways, signaling pathways were elevated (**Figure 9K**). This highlights the role of IL10 in immune regulation and inflammatory response.

### Molecular docking analysis

Through correlation analysis, Interleukin-10 (IL-10) was identified as an inflammatory cytokine associated with both IE and sepsis. The protein structure of IL-10 was obtained from the PDB database, and the structures of four small molecule drugs—resveratrol, curcumin, matrine, and taurine—were retrieved from the PubChem database.

**Figure 10:**
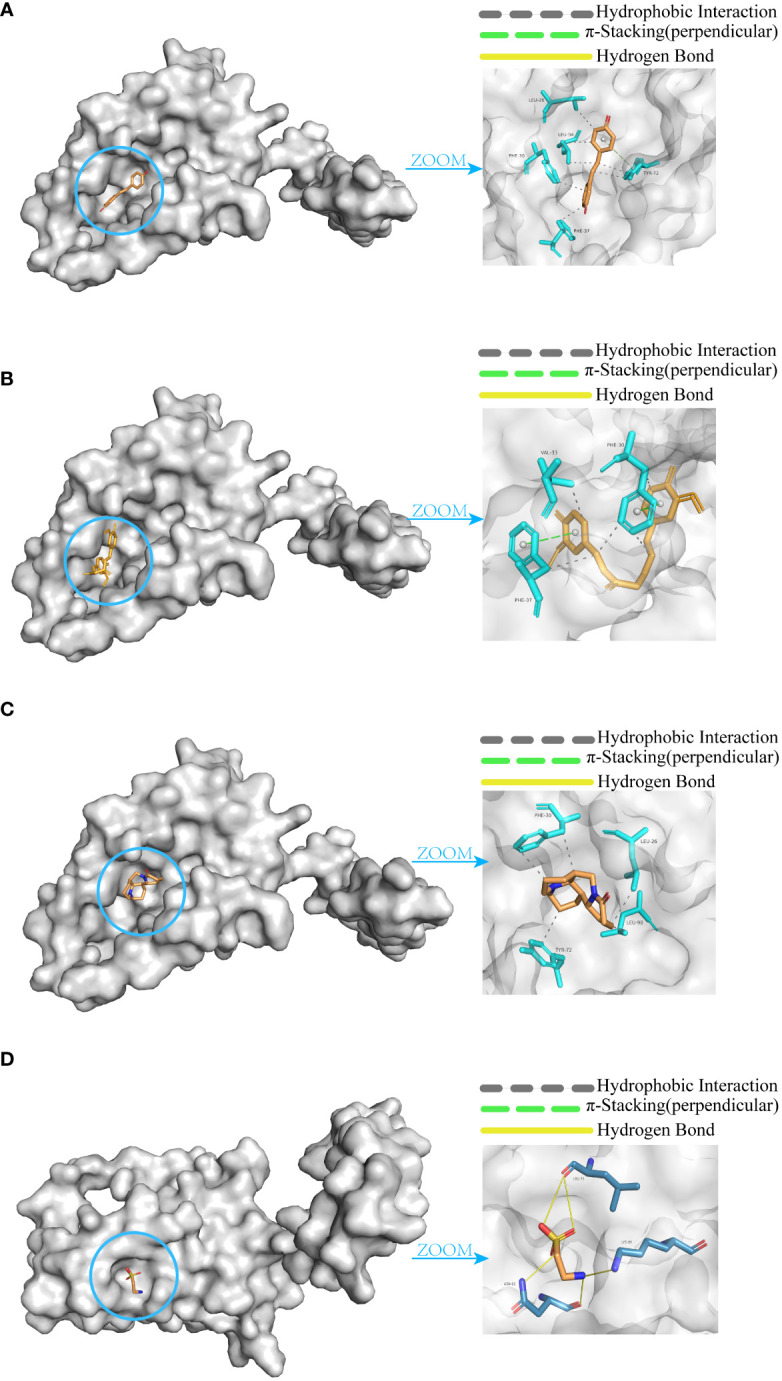
Molecular docking of IL-10 with small molecules. **(A)** Resveratrol. **(B)** Curcumin. **(C)** Matrine. **(D)** Taurine.

Molecular docking was performed between IL-10 and these four small molecules. The results revealed that resveratrol (**Figure 10A**), curcumin (**Figure 10B**), and matrine (**Figure 10C**) share similar binding pockets and have overlapping binding sites on IL-10. On the other hand, taurine (**Figure 10D**) exhibits a different binding pocket on IL-10 and has considerably different binding sites compared to the other three molecules.

The affinity and docking sites for each small molecule with IL-10 are listed in **Table 2**. Upon comparison, it was found that resveratrol, curcumin, and matrine exhibit good docking results with IL-10 (Affinity < -5.0 kcal/mol). In contrast, the docking effectiveness of taurine with IL-10 is not as promising as the other three small molecules.

**Table 2:** Molecular docking data.

Small molecule	Affinity(kcal/mol)	Binding site
Resveratrol	-6.3	LEU-26,PHE-30,PHE-37,TYR-72,LEU-94
Curcumin	-6.9	PHE-30,VAL-33,PHE-37
Matrine	-6.4	LEU-26,PHE-30,TYR-72,LEU-98
Taurine	-3.3	LEU-73,ASN-92,LYS-99

### qRT-PCR validation

**Figure 11:**
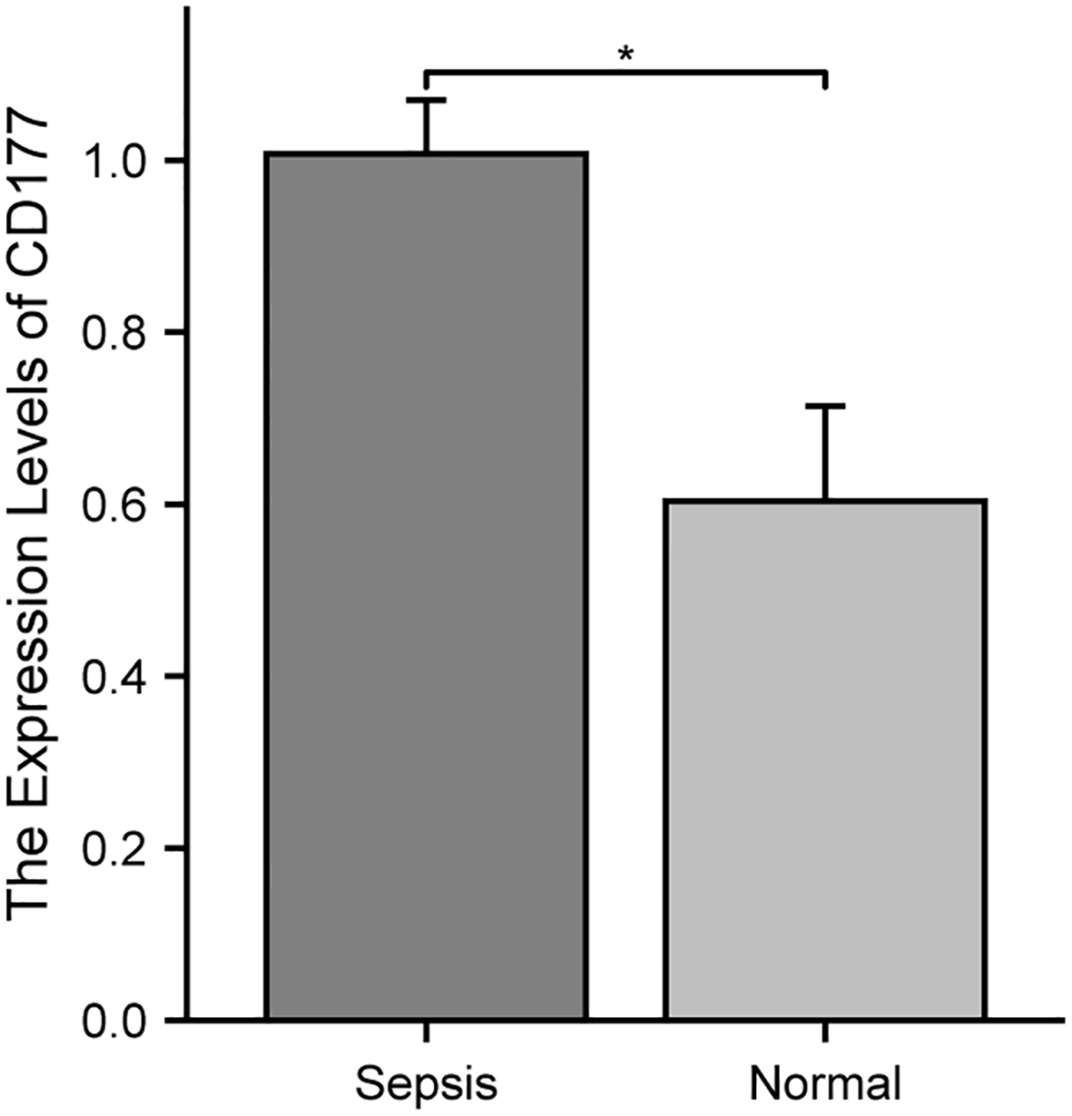
Validation of the reliability of the CD177 as biomarker by qRT-PCR. * P<0.05, ** P<0.01, *** P<0.001, ns: not significance.

To further validate the differential expression of the biomarker CD177 identified in our single-cell RNA sequencing, we conducted independent experiments using the qRT-PCR method. The results (**Figure 11**) clearly show that the expression level of CD177 is significantly higher in sepsis samples than in normal samples. This finding is consistent with our previous observation of CD177 overexpression based on data analysis, further confirming the key role of CD177 in sepsis.

## Discussion

Infective endocarditis (IE) shares similar pathogenic mechanisms with sepsis and may be caused by infections from bacteria such as Staphylococcus and Streptococcus (15–17). In addition to exhibiting invasive and destructive properties on the surface of the endocardium, the pathogenic bacteria of IE may also lead to systemic infections, excessive activation of the immune system through the secretion of exotoxins, and the development of sepsis complications. Therefore, early diagnosis of IE and sepsis is of great significance in preventing complications and reducing mortality.

In this study, cutting-edge bioinformatics analysis methods were employed to identify 76 key target genes associated with IE and sepsis. These genes play significant roles in regulating protease activity, immune cell activation, molecular interactions, as well as Th1 and Th2 cell differentiation. Previous studies have shown that extracellular vesicles from non-immune cells can promote the differentiation of Th1/Th2 cells in the late stage of sepsis (18). Specifically, Th1 cells can produce IFN-γ, IL-2, and TNF-β, which activate macrophages and are responsible for cell-mediated immunity and phagocyte-dependent protective responses. Meanwhile, Th2 cells enhance antibody production, activate eosinophils, and inhibit several macrophage functions through the production of IL-4, IL-5, IL-10, and IL-13, providing phagocyte-independent protective responses (19). It is worth noting that studies have found an upregulation of Th1 cells and a downregulation of Th2 cells in patients with sepsis (20). This finding suggests that the immune response in sepsis patients may primarily rely on phagocyte-dependent protective responses. Through these discoveries, we can gain a deeper understanding of the molecular mechanisms of IE and sepsis, providing important evidence for future clinical treatments.

Through scRNA-seq and immune cell infiltration-related analysis of sepsis patients, we have observed a clear decrease in the number of B cells and T cells, while the number of monocytes has correspondingly increased. This data is consistent with previous studies that have demonstrated impaired B cell maturation in the sepsis environment (21), which is not only a key factor in the reduction of B cell numbers but also closely associated with poor prognosis of the disease. It is worth noting that the decrease in T cell numbers is not solely caused by a single factor. According to literature reports (22), sepsis not only induces T cell apoptosis but also continuously impairs the function of the remaining T cells. This complex impact may further weaken the overall efficiency of the immune system. At the same time, monocytes play an indispensable role in sepsis (23). This series of changes in the immune microenvironment in patients with sepsis is likely to dysfunction the immune system and lead to immunosuppression (24), so that pathogens invading patients cannot be eliminated rapidly, making it difficult to treat inflammation and even leading to new secondary infections. In summary, this series of immune cell changes provides a new framework for understanding the complex course and poor prognosis of sepsis, and also points out possible research directions for future sepsis treatment.

Through analysis of immune infiltrating cells between two clusters in sepsis, we observed significant differences in the abundance and expression levels of infiltrating immune cell populations, revealing potential differences in inflammatory responses and treatment strategies between the two clusters of sepsis. It is worth noting that Th1 cells are significantly upregulated in sepsis cluster 2 compared to sepsis cluster 1, and this excessive Th1 cell expression may disrupt immune balance, leading to tissue damage. This observation suggests that the inflammatory response in sepsis cluster 2 may be more intense, potentially accompanied by more severe clinical symptoms and poorer treatment outcomes. Therefore, different treatment strategies may need to be formulated based on the immune response characteristics of different clusters in order to more effectively control the progression and impact of sepsis.

By applying machine learning methods, this study has identified four potential diagnostic biomarkers associated with Infective Endocarditis (IE) and Sepsis. Firstly, S100A12 has been identified as a key molecule, playing a crucial role in the immune and inflammatory responses in IE patients (7), while primarily released at the infection site in sepsis patients (25). Additionally, CD177 showed significantly increased expression in sepsis patients (26), whereas IRAK3 is associated with the immune suppression stage (27). Beyond these confirmed genes, this study also discovered three potential diagnostic genes for IE (CD177, IRAK3, RNASE2) and one potential diagnostic gene for sepsis (RNASE2) for the first time. Analysis of both microarray data, RNA-seq data and scRNA-seq data demonstrated consistent expression levels of the mentioned genes, providing further validation of the results. Notably, CD177 not only holds high diagnostic value for both IE and sepsis but also achieves good classification results in two distinct sepsis clusters with significant differences in immune cell populations. Meanwhile, based on the DEGs from two sepsis clusters, enrichment analysis reveals distinct pathways associated with CD177. It is evident that these two sepsis clusters exhibit variations in their roles in immune and inflammatory responses. For instance, one sepsis cluster may be more sensitive to infection or inflammation, displaying a more robust immune response, while the other cluster may exhibit a weaker response to the same stimuli. These findings not only unveil potential development mechanisms for IE and sepsis but also provide essential new leads for the study of their immune responses and treatment strategies.

Further analysis suggests that Interleukin-10 (IL-10), as an anti-inflammatory cytokine associated with both Infective Endocarditis (IE) and sepsis, could potentially serve as a common therapeutic target protein for both diseases. Firstly, from previous research, we understand that IL-10 can act on antigen-presenting cells to inhibit Th1 cell cytokine production (28), thereby reducing inflammatory responses and tissue damage. In light of this mechanism, this study particularly focused on small molecule drugs with anti-inflammatory properties: resveratrol, curcumin, matrine, and taurine. Among these, resveratrol and curcumin have been confirmed to upregulate IL-10 expression and production, hence having anti-inflammatory effects (29, 30). Molecular docking results from this study further confirmed the strong binding of resveratrol and curcumin with IL-10. For matrine and taurine, this study found that taurine docked poorly with IL-10, whereas matrine was molecularly conformationally stable and had the same binding pocket as resveratrol and curcumin on IL-10 with similar binding sites. Therefore, it is speculated that matrine may also exert anti-inflammatory effects by targeting IL-10 in IE and sepsis. However, it is worth noting that due to differential expression of IL-10 between sepsis clusters, with sepsis cluster 1 showing significantly higher IL-10 expression than sepsis cluster 2, the potential therapeutic effects of matrine may vary between different sepsis clusters, with more pronounced effects in cluster 1 with high IL-10 expression. These findings provide a new mechanistic understanding and potential drug candidates for anti-inflammatory treatment of IE and sepsis.

Of course, this study inevitably carries some limitations. Firstly, while we obtained a certain dataset from the GEO database, we were restricted by the data volume, and thus, we could only validate the diagnostic potential of the identified genes in IE using the available dataset. For a more in-depth understanding of the molecular mechanisms behind these diagnostic genes and to assess the association between clinical data and diagnostic genes, future work will require the integration of additional external datasets, biological experimental studies, and clinical trials. Secondly, the potential therapeutic value of Interleukin-10 (IL-10) proposed in this study for IE and sepsis, as well as the mode of action of matrine, although theoretically grounded, indeed demands further empirical research for validation and deeper exploration. Specifically, the specific mechanisms of action of matrine in controlling IE and sepsis, especially in different sepsis clusters, will require more experimental support. In summary, this study provides a preliminary outlook on common biomarkers for IE and sepsis and offers crucial theoretical and empirical evidence for the application of matrine in treating these diseases. However, further extensive and in-depth research is needed to refine and support these propositions.

## Conclusions

In summary, this study delves into the shared pathogenic mechanisms between Infective Endocarditis (IE) and sepsis, uncovering common biomarkers and potential therapeutic targets. Through an analysis of shared target genes, scRNA-seq data, and immune factor profiling, we reveal the critical roles of immune cells such as Th1 cells, B cells, T cells, IL-10, immune cells and cytokines in the activation and regulation of these two diseases. Particularly, IL-10, as a vital anti-inflammatory cytokine, is identified as a common therapeutic target protein, and potential therapeutic drug matrine is discovered through molecular docking. Further analysis also demonstrates differences between subtypes of sepsis, including significant variations in the abundance and expression levels of immune cell populations, which may lead to distinct inflammatory responses and treatment strategies. These findings not only lay the foundation for more precise sepsis diagnosis and subtyping but also open up new possibilities for personalized treatment tailored to different subtypes. While this study is limited by data volume and experimental validation, it undoubtedly provides a fresh perspective and direction for the early diagnosis and treatment of IE and sepsis, particularly in exploring new potential diagnostic biomarkers and therapeutic targets. Future research will require more extensive datasets and experimental validation to establish these findings and delve deeper into potential molecular mechanisms, aiming to provide a more robust foundation for clinical diagnosis and treatment.

## Data availability statement

The datasets presented in this study can be found in online repositories. The names of the repository/repositories and accession number(s) can be found in the article/**Supplementary Material**.

## Ethics statement

The studies involving human participants were reviewed and approved by the Committee on Human Research of Maternal and Child Health Hospital of Hubei Province (2023IEC055). Written informed consent to participate in this study was provided by the patient legal guardian.

## Author contributions

DC: Supervision, Validation, Writing – review & editing. CY: Data curation, Formal analysis, Writing – original draft. HZ: Methodology, Software, Writing – review & editing. JY: Data curation, Formal analysis, Software, Writing – review & editing. SJ: Funding acquisition, Project administration, Resources, Writing – review & editing.
