# Supplementary material for: Elucidating common pathogenic transcriptional networks in infective endocarditis and sepsis: integrated insights from biomarker discovery and single-cell RNA sequencing
Source: Front Immunol. 2024 Jan 25;14:1298041. doi: 10.3389/fimmu.2023.1298041 (PMC10851146; doi:10.3389/fimmu.2023.1298041)
Supplement: Supplementary file 1 [file DataSheet_1.docx]

**
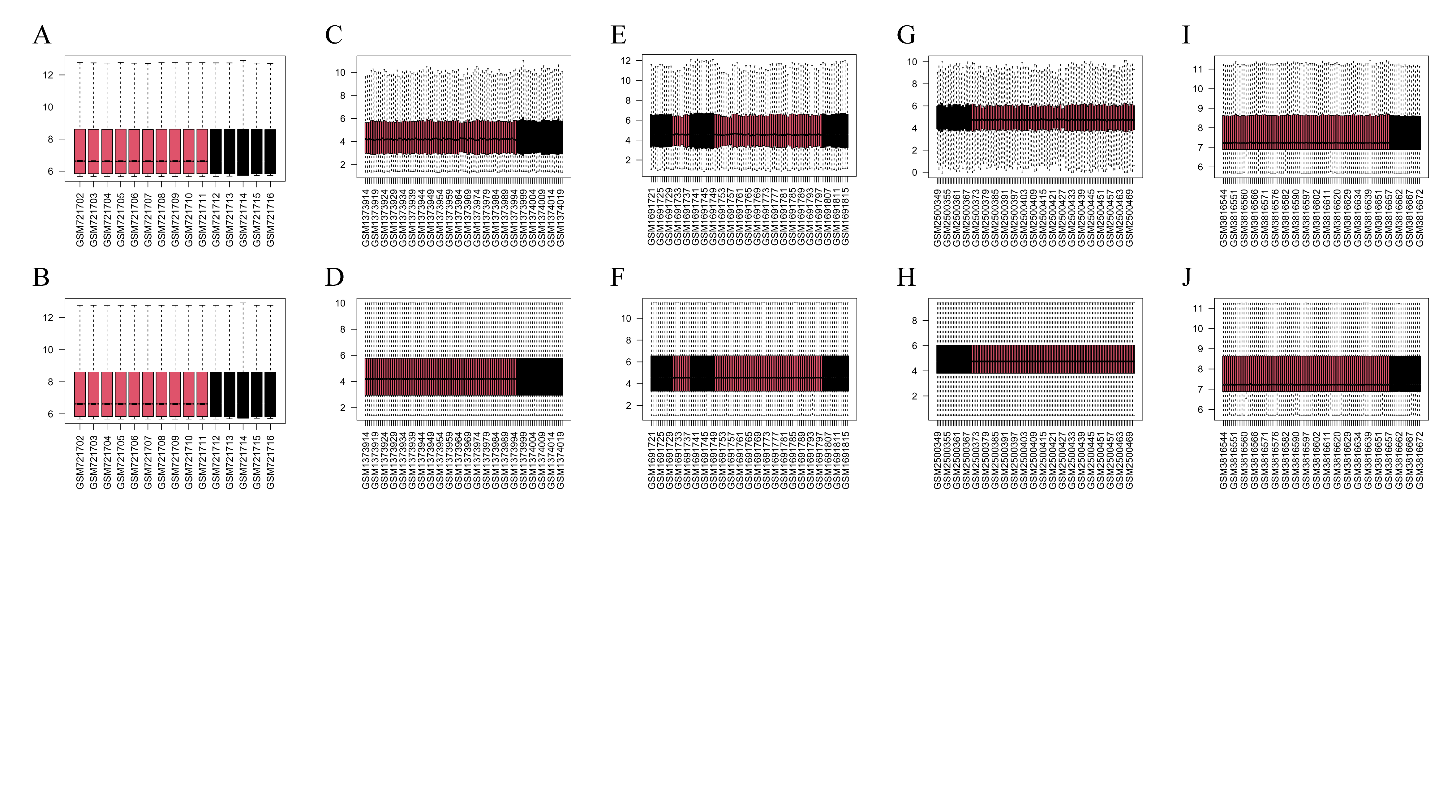
**

**Supplementary Figure 1**

The box plots before and after microarray data normalization, red-patient, black-healthy control. **(A)** Before normalization of GSE29161. **(B)** After normalization of GSE29161. **(C)** Before normalization of GSE57065. **(D)** After normalization of GSE57065. **(E)** Before normalization of GSE69063. **(F)** After normalization of GSE69063. **(G)** Before normalization of GSE95233. **(H)** After normalization of GSE95233. **(I)** Before normalization of GSE131761. **(J)** After normalization of GSE131761.

**
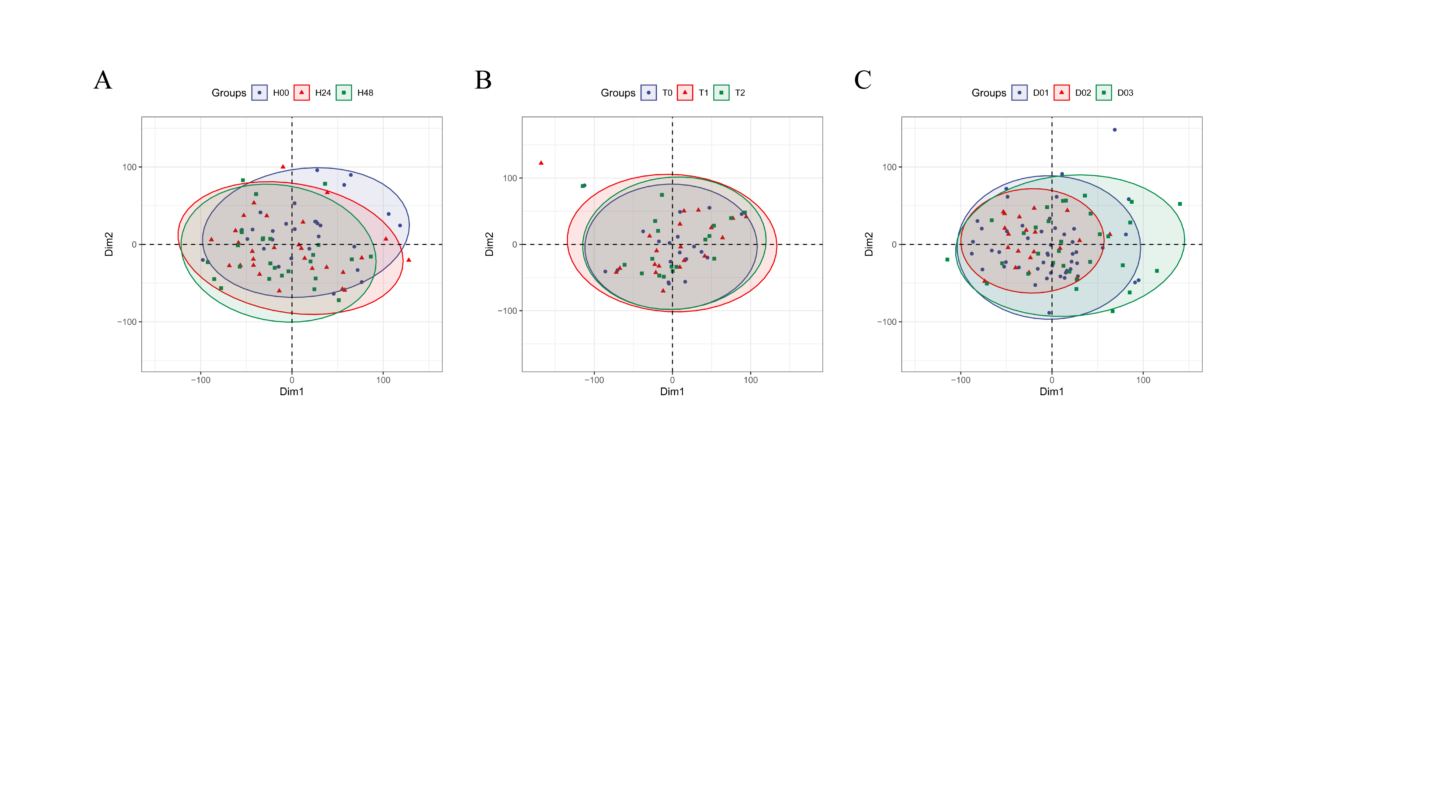
**

**Supplementary Figure 2**

Principal Component Analysis of sepsis patient samples at different time points. **(A)** GSE57065, H00 - 30 minutes post-diagnosis, H24 - 24 hours post-diagnosis, H48 - 48 hours post-diagnosis. **(B)** GSE69063, T0 - upon arrival at the emergency department, T1 - one hour after arrival, T2 - 3 hours after arrival. **(C)** GSE95233, D01 - on the first day post-diagnosis, D02 - on the second day post-diagnosis, D03 - on the third day post-diagnosis.

**
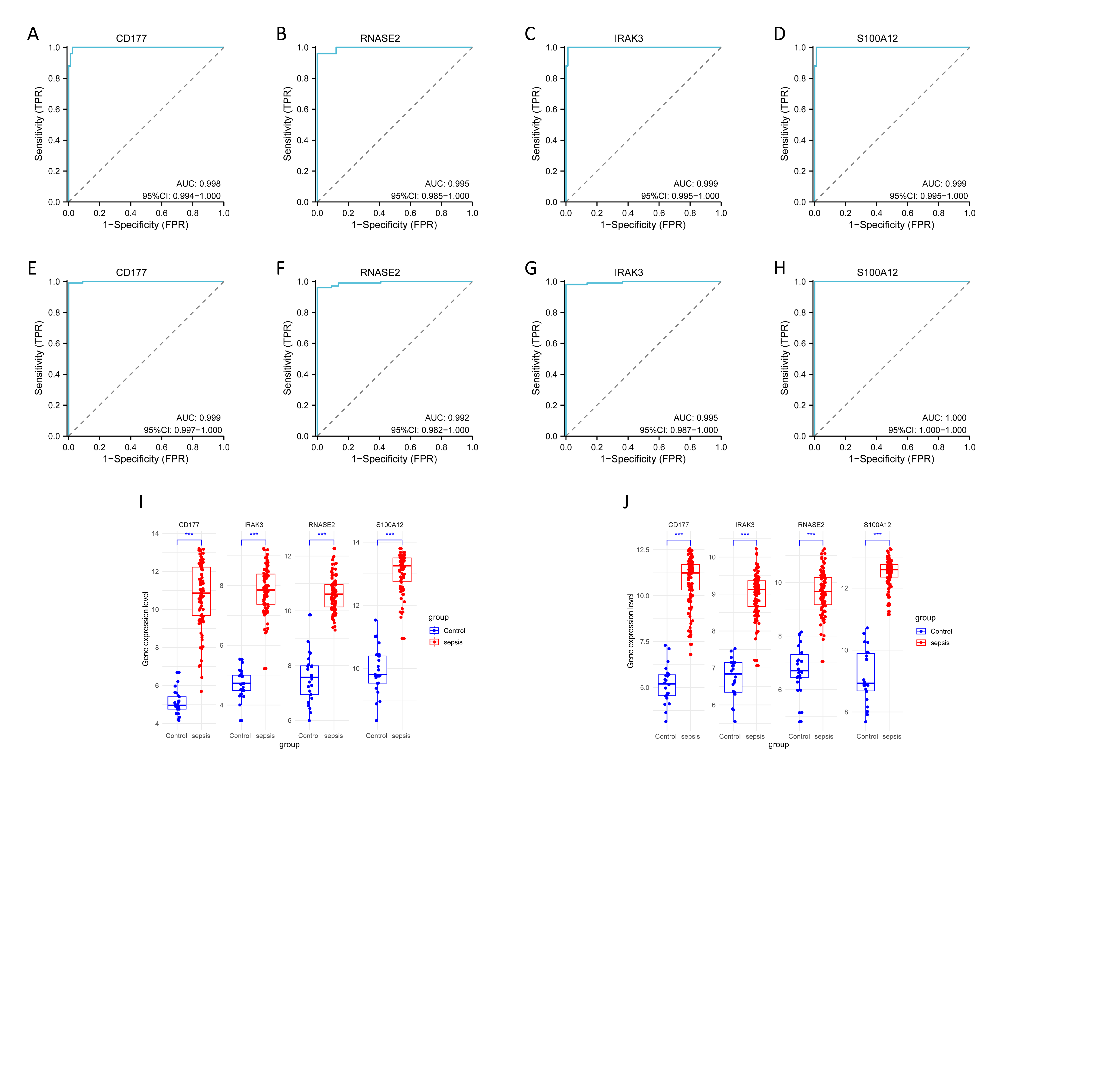
**

**Supplementary Figure 3**

Capacity assessment of key biomarkers in sepsis analysis datasets. **(A-D)** ROC curve for GSE57065. **(E-H)** ROC curve for GSE95233. **(I)** Expression levels of diagnostic genes for GSE57065. **(J)** Expression levels of diagnostic genes for GSE95233.

**
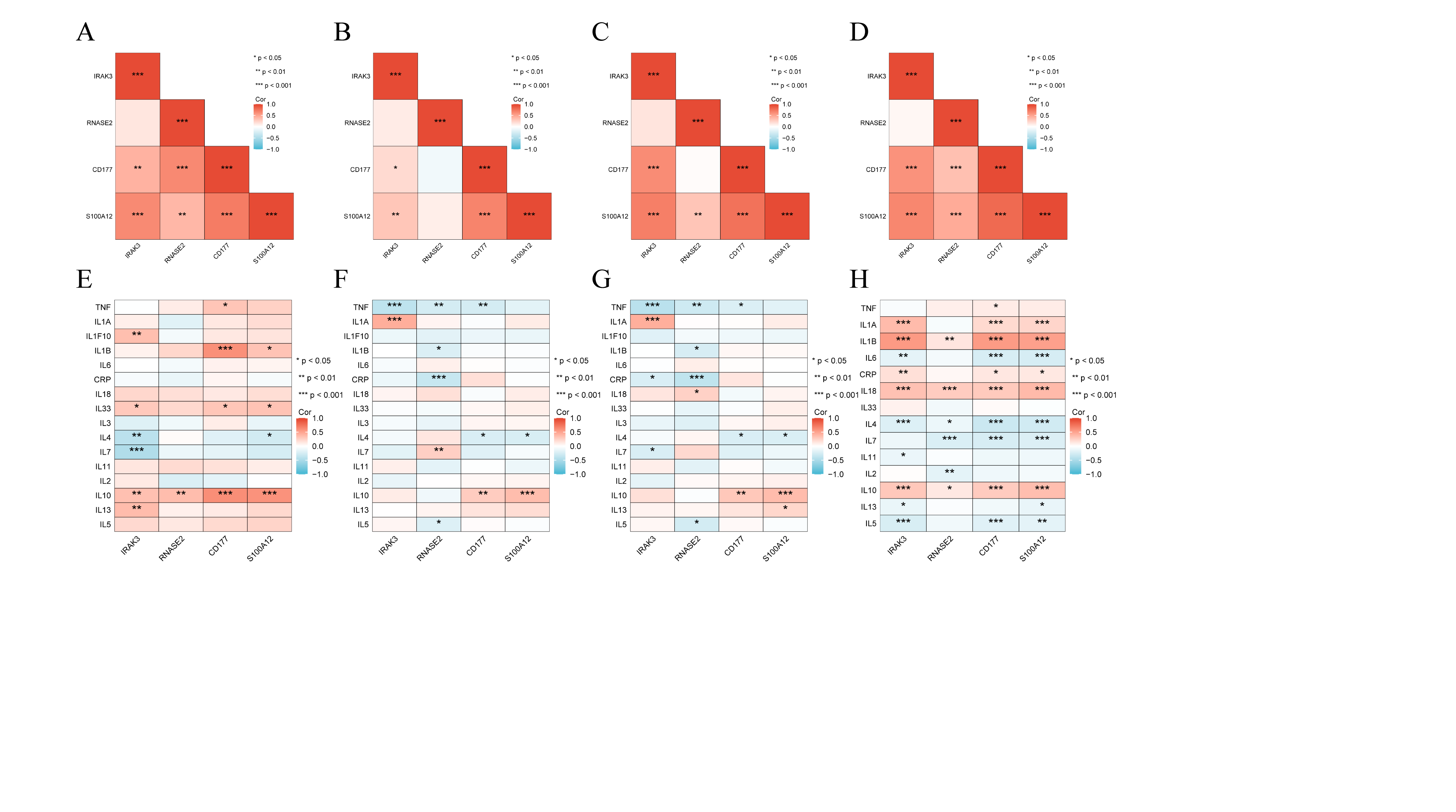
**

**Supplementary Figure 4**

The correlation within diagnostic genes and their correlation with inflammatory factors. **(A-D)** The correlation within diagnostic genes, A-GSE69063, B-GSE95233, C-GSE131761, D-GSE185263. **(E-H)** The correlation between diagnostic genes and inflammatory factors.

**Supplementary Table 1**

Quality screening criteria for each sample of scRNA-seq data

| **Group** | **Sample** | **Screening criteria** | **Cell number** |
| --- | --- | --- | --- |
| Sepsis | Sepsis1 | 300<nFeature_RNA<2500,nCount_RNA<7500,  2<percent.mt<7.5 | 701 |
|  | Sepsis2 | 500<nFeature_RNA<2000,nCount_RNA<6000,  1<percent.mt<7.5 | 1537 |
|  | Sepsis3 | 300<nFeature_RNA<2500,nCount_RNA<8000,  1<percent.mt<15 | 7094 |
|  | Sepsis4 | 300<nFeature_RNA<3000,nCount_RNA<10000,  2<percent.mt<10 | 5255 |
| HC | HC1 | 300<nFeature_RNA<2500,nCount_RNA<7500,  1<percent.mt<10 | 1051 |
|  | HC2 | 200<nFeature_RNA<2000,nCount_RNA<6000,  percent.mt<7.5 | 709 |
|  | HC3 | 200<nFeature_RNA<2500,nCount_RNA<7500,  2<percent.mt<8 | 8084 |
|  | HC4 | 200<nFeature_RNA<3000,nCount_RNA<7500,  2.5<percent.mt<7.5 | 4157 |
|  | HC5 | 500<nFeature_RNA<3000,nCount_RNA<10000,  2.5<percent.mt<10 | 3881 |

**Supplementary Table 2**

Primer sequence

| **Primer name** | **Sequences** | **Extended Clip（bp）** |
| --- | --- | --- |
| CD177-F | GTGCCCAGTCTGCTTGTCT | 211 |
| CD177-R | GTTCTCAGTCATACCCACGG |  |
| GAPDH-F | GGGAAACTGTGGCTTGAT | 299 |
| GAPDH- R | GAGTGGGTGTCGCTGTTGA |  |
